# Neuroprotective Effects of a Standardized Flavonoid Extract from Safflower against a Rotenone-Induced Rat Model of Parkinson’s Disease

**DOI:** 10.3390/molecules21091107

**Published:** 2016-08-24

**Authors:** Nuramatjan Ablat, Deyong Lv, Rutong Ren, Yilixiati Xiaokaiti, Xiang Ma, Xin Zhao, Yi Sun, Hui Lei, Jiamin Xu, Yingcong Ma, Xianrong Qi, Min Ye, Feng Xu, Hongbin Han, Xiaoping Pu

**Affiliations:** 1 Department of Molecular and Cellular Pharmacology, School of Pharmaceutical Sciences, Peking University, Beijing 100191, China; nuramatjan817@bjmu.edu.cn (N.A.); rrt874609@163.com (R.R.); maxiang19910215@163.com (X.M.); guoguo2891@126.com (X.Z.); sunyi@bjmu.edu.cn (Y.S.); leihui2006sdu@sina.cn (H.L.); zeroby@126.com (J.X.); 2 State Key Laboratory of Natural and Biomimetic Drugs, Peking University, Beijing 100191, China; Elshat.shevket@gmail.com (Y.X.); mayingcong123@163.com (Y.M.); qixr@bjmu.edu.cn (X.Q.); yemin@bjmu.edu.cn (M.Y.); xufeng_pharm@163.com (F.X.); 3 Department of Radiology, Peking University Third Hospital, Beijing100191, China; ldy9408@sohu.com; 4 Department of Radiology, Dongying People’s Hospital of Shandong, Dongying 257091, China; 5 Department of Molecular and Cellular Pharmacology, School of Basic Medical Sciences, Peking University, Beijing100191, China; 6 Beijing Key Lab of MRI Device and Technique, Peking University Third Hospital, Beijing 100191, China

**Keywords:** safflower flavonoid extract, Parkinson’s disease, DA, MRI

## Abstract

Parkinson’s disease (PD) is a major age-related neurodegenerative disorder characterized by the loss of dopaminergic neurons in the substantia nigra par compacta (SNpc). Rotenone is a neurotoxin that is routinely used to model PD to aid in understanding the mechanisms of neuronal death. Safflower (*Carthamus tinctorius*. L.) has long been used to treat cerebrovascular diseases in China. This plant contains flavonoids, which have been reported to be effective in models of neurodegenerative disease. We previously reported that kaempferol derivatives from safflower could bind DJ-1, a protein associated with PD, and that a flavonoid extract from safflower exhibited neuroprotective effects in the 1-methyl-4-phenyl-1,2,3,6-tetrahydropyridine-induced mouse model of PD. In this study, a standardized safflower flavonoid extract (SAFE) was isolated from safflower and found to primarily contain flavonoids. The aim of the current study was to confirm the neuroprotective effects of SAFE in rotenone-induced Parkinson rats. The results showed that SAFE treatment increased body weight and improved rearing behavior and grip strength. SAFE (35 or 70 mg/kg/day) treatment reversed the decreased protein expression of tyrosine hydroxylase, dopamine transporter and DJ-1 and increased the levels of dopamine and its metabolite. In contrast, acetylcholine levels were decreased. SAFE treatment also led to partial inhibition of PD-associated changes in extracellular space diffusion parameters. These changes were detected using a magnetic resonance imaging (MRI) tracer-based method, which provides novel information regarding neuronal loss and astrocyte activation. Thus, our results indicate that SAFE represents a potential therapeutic herbal treatment for PD.

## 1. Introduction

Neurodegenerative disorders, such as Parkinson’s disease (PD) and Alzheimer’s disease (AD), affect millions of patients at ever growing numbers in aging societies, and no curative treatments exist [1]. PD is the second most common chronic and progressive neurodegenerative disorder after AD [2]. Disease onset in PD is variable and can be observed in younger, middle-aged or elderly patients, although this disease is most commonly observed in adults over 60 years of age [3,4]. PD is clinically characterized by symptoms such as akinesia, rigidity, bradykinesia, resting tremor, postural instability and balance, and sensory-motor integration deficits [5,6,7]. Progressive loss of dopaminergic neurons in the substantia nigra par compacta (SNpc) and subsequent depletion of dopamine (DA) in the striatum, which is the main projection area of the substantia nigra (SN), is the primary cause of PD symptoms [8]. Various environmental and genetic factors have been suggested, but the etiology of PD remains largely unknown [3]. Epidemiological evidence has indicated that pesticides and other environmental toxins may play a role in the development of idiopathic PD [9]. Rotenone is a pesticide and toxin that can be used to induce PD in an animal model [10]. In rats, rotenone reproduces many key pathological features of PD such as oxidative damage, α-synuclein aggregation [11], oxidative stress-induced striatal dopaminergic terminal degeneration [12], selective nigrostriatal loss, cognitive deficits and depression-like behavior [13]. Currently, DA supplement therapy is a standard treatment for PD. Although Madopar^®^ (levodopa + benserazide) is the gold standard treatment, long-term use of this drug can cause adverse symptoms [14].

Flavonoids are the active components of many medicinal herbs and exert many health-related properties. Pharmacological studies indicate that extracts from safflower (*Carthamus tinctorius.* L*.*), a member of the Compositae family that is natively distributed over Asia and some African countries, show a number of effects including neuroprotective [15], cardioprotective [16], anti-fibrotic [17], anti-coagulation and anti-thrombotic [18], anti-aging [19], vasodilation and anti-hypertensive [20], and anti-oxidative [21] properties.

In earlier studies, our group confirmed that compounds isolated from safflower such as kaempferol 3-*O*-rutinoside (K3R) and anhydrosafflor yellow B (AYB) could reduce the levels of hydrogen peroxide (H_2_O_2_)-induced reactive oxygen species in PC12 cells. DJ-1 (also known as PARK7), a causative gene product from a familial form of PD, plays a role in anti-oxidative stress responses, and loss of DJ-1 function is thought to result in the onset of PD. Additional work from our group also found that K3R and AYB were found to specifically bind to the C106 region by using a quartz crystal microbalance. K3R levels was diminished in cells transfected with siRNA targeting DJ-1, indicating DJ-1-dependent activity of K3R and DJ-1-independent activity of AYB [22]. Furthermore, flavonoid extracts from safflower have been shown to exert a neuroprotective effect against the 1-methyl-4-phenyl-1,2,3,6-tetrahydropyridine (MPTP)-induced mouse model of PD [23].

Brain tissue is comprised of two components: cellular elements (neurons and glial cells) and the space between the cellular elements—the extracellular space (ECS) [24]. Neurons are bathed by interstitial fluid (ISF) within the brain ECS, which forms the microenvironment of the central nervous system (CNS) [25,26]. Thus, changes in ECS diffusion parameters may be a key factor in identifying the mechanisms and pathologic changes that underlie PD and may reveal improved therapeutic approaches for the treatment of PD.

As previously described, rotenone is a chemical that can cause PD, but there is no research regarding the use of a standardized flavonoid extract of safflower (SAFE) for treatment in a rotenone-induced PD animal model. In this study, we investigated the neuroprotective effects of a standardized flavonoid extract of safflower, using K3R and AYB levels as quality standards, in a rotenone-induced PD rat model.

## 2. Results

### 2.1. Qualitative Quantitative Analyses of SAFE Components

**Figure 1:**
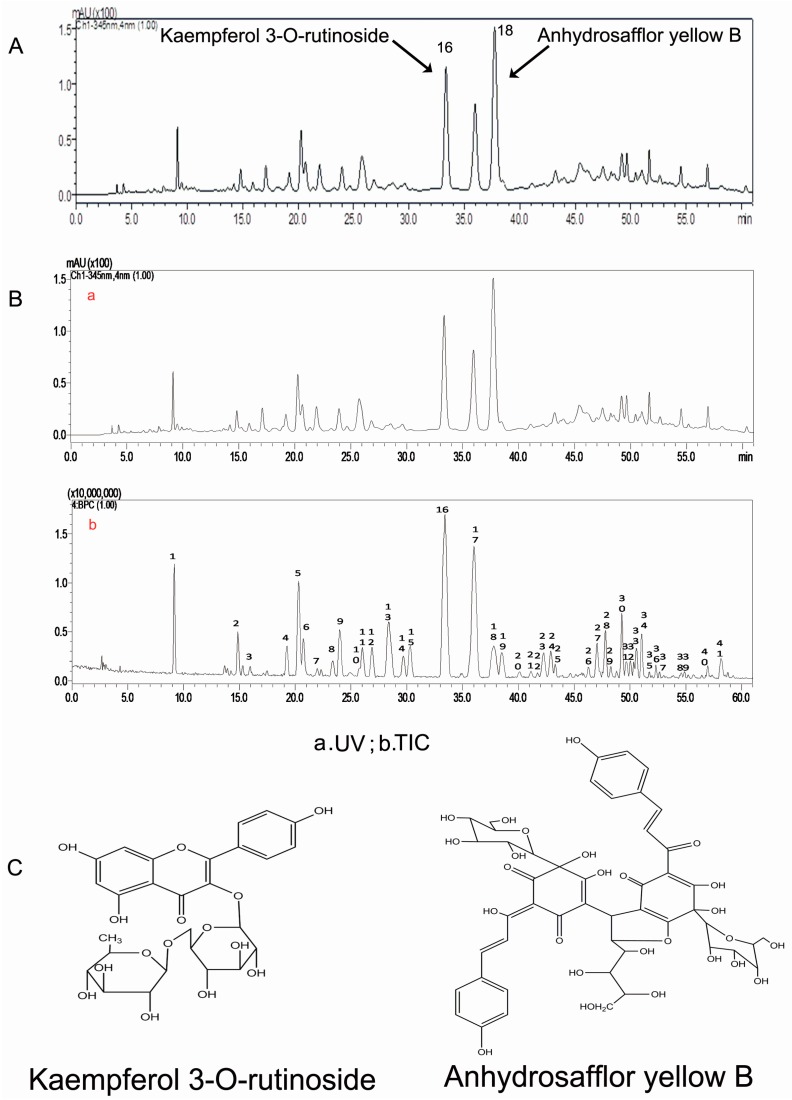
(**A**) The HPLC chromatogram of SAFE; (**B**) The HPLC chromatogram at 345 nm of SAFE; negative ion base peak chromatogram of SAFE; (**C**) Chemical structures of kaempferol 3-*O*-rutinoside and anhydrosafflor yellow B.

High-performance liquid chromatography (HPLC) analysis showed that the 30% ethanol elutate of safflower contained K3R and AYB, as shown in the standardized HPLC chromatogram of SAFE (Figure 1A,C). Our results indicated that the primary components were flavonoids, which accounted for 70.75% of the total number of components in SAFE. K3R and AYB were representative compounds of glycoside and chalcone flavonoids (two flavonoid subclasses), respectively. According to the liquid chromatography/mass spectrometry (LC/MS) analyses (Figure 1B, Table 1), the extract was comprised of approximately 7.83% K3R (No. 16) and 7.97% AYB (No. 18) along with other less prevalent flavonoids, including anhydrosafflor yellow A (AYA) (No. 1), quercetin 3-*O*-rutinoside (rutin) (Q3R) (No. 4), isorhamnetin methylpentosyl hexoside (No. 17), and 4′,5-dihydroxyl-6-*O*-glucopyranosyl flavanone (4D6GF) or its isomer (No. 6) [23].

### 2.2. Changes in Body Weight and Behavior

**Figure 2:**
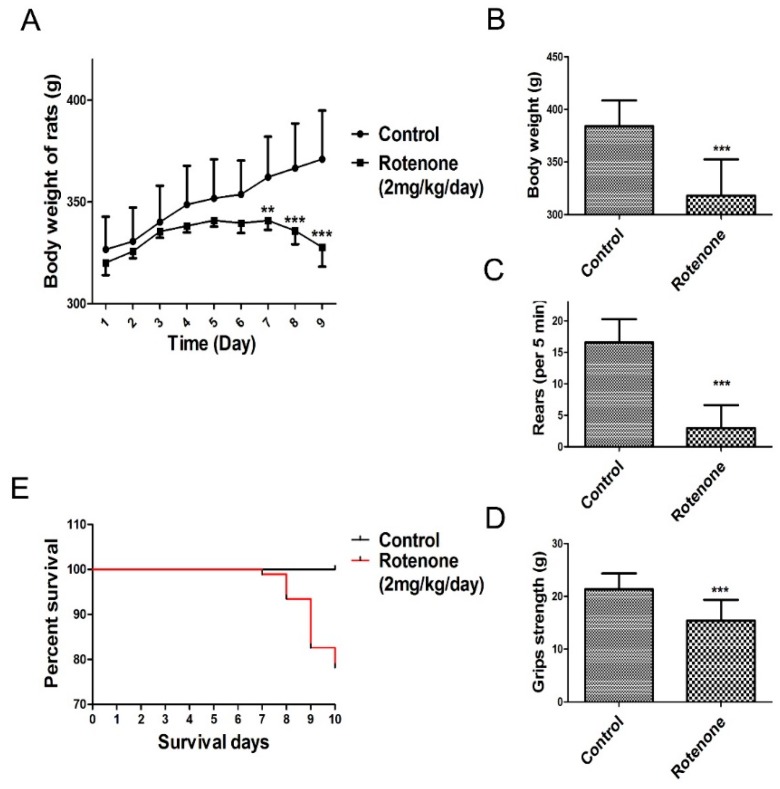
Changes in body weight and behavioral parameters of the PD model. The parameters are presented as the mean ± SD, * compared to control, (** *p* < 0.01, *** *p* < 0.001, rotenone: *n* = 92, control: *n* = 10). (**A**) Body weight change during rotenone treatment from day 1 to day 9; (**B**) Day 10 body weight change during rotenone treatment; (**C**) Rearing behavior result after 10 days of rotenone treatment; (**D**) Grip strength result after 10 days of rotenone treatment; (**E**) Survival of during 10 days rotenone treatment.

Daily subcutaneous injections of rotenone elicited a progressive loss in body weight and behavioral deficits. The present study observed that rats in the rotenone-treated group consumed less food and water compared to rats in the control group. As shown in Figure 2A (body weight change during rotenone treatment from day 1 to day 9), the body weight of the rats was markedly decreased following rotenone treatment for 7 days compared to the control group. In addition, a significant decrease in body weight was observed in rotenone‑treated rats at 10 days following rotenone treatment compared to the control group (*p* < 0.001, Figure 2B).

During the experimental procedure, none of the rats in the control group died. In contrast, as shown in Figure 2E, 20 rats in the rotenone-treated group succumbed over the course of their injection regimen (1, 5, 10 and 4 rats succumbed at days 7, 8, 9 and 10, respectively, after the initiation of rote-none treatment).

Results from the rearing behavior and grip strength tests showed that, after 10 days of rotenone treatment, rotenone-treated rats presented a significant decrease in rearing behavior and grip strength compared to the control group (*p* < 0.001, Figure 2C,D).

After 10 days of rotenone treatment, the rats were randomly divided into four groups based on body weight, rearing behavior and grip strength and reclassified as follows: rotenone group, low-dose SAFE (35 mg/kg/day) group, high-dose SAFE (70 mg/kg/day) group and Madopar^®^ (50 mg/kg/day) group.

**Figure 3:**
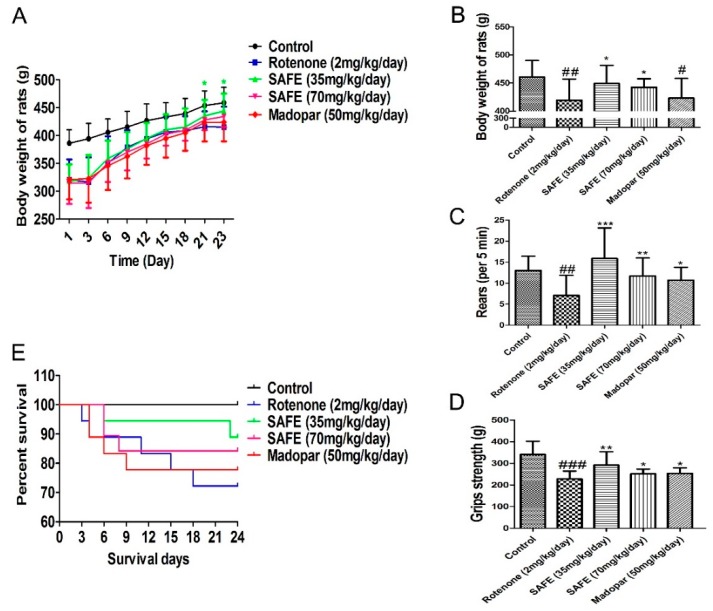
Changes in body weight and behavioral parameters during SAFE treatment. (**A**) Body weight change during the SAFE treatments from day 1 to day 23; (**B**) Body weight change during the SAFE treatments day 24; (**C**) Rearing behavior result after 24 days of SAFE treatments; (**D**) Grip strength result after 24 days of SAFE treatments; (**E**) Survival of during 24 days SAFE treatments. The parameters are presented as the mean ± SD, (**^#^** compared to control, **^#^** *p* < 0.05, **^##^** *p* < 0.01, **^###^** *p* < 0.001; * compared to rotenone * *p* < 0.05, ** *p* < 0.01, *** *p* < 0.001) (control *n* = 10, other group *n* = 18).

Rats in the SAFE and Madopar^®^ groups consumed more food and water compared to the rats in the rotenone group. As shown in Figure 3A (body weight change during the SAFE and Madopar^®^ treatments from day 1 to day 23), the body weight of the low-dose SAFE (35 mg/kg/day) group rats was markedly increased following treatment from day 21 to day 23 compared to rotenone treatment (*p* < 0.05). In addition, a significant increase was observed in the body weight of SAFE-treated rats at 24 days following SAFE (35 or 70 mg/kg/day) treatment compared to the rotenone group (both *p* < 0.05, Figure 3B). No significant increase was observed in the body weight of Madopar^®^-treated rats at 24 days following Madopar^®^ treatment compared to the rotenone group (*p* > 0.05, Figure 3B).

Results from the rearing behavior and grip strength analyses showed that after 24 days of treatment, the rearing behavior and grip strength in the SAFE and Madopar^®^-treated group significantly increased compared to the rotenone group, low-dose SAFE treated group (both *p* < 0.001, and *p* < 0.01), high-dose SAFE treated group (both *p* < 0.01, and *p* < 0.05) and Madopar^®^-treated (both *p* < 0.05, Figure 3C,D) respectively.

During the secondary phase of the experimental procedure, none of the rats in the control group died. By contrast, as shown in Figure 3E, 5 rats in the rotenone-treated group (1 each on days 3, 4, 11, 15 and 18); 2 rats in the low-dose SAFE-treated group (1 each on days 6 and 23); 3 rats in the high-dose SAFE-treated group (2 and 1 rats on days 6 and 8, respectively) and 4 rats in the Madopar^®^-group (2, 1 and 1 rats on days 4, 6 and 9, respectively, after the initiation of saline treatment) succumbed to their ailments.

### 2.3. Western Blot Analysis

**Figure 4:**
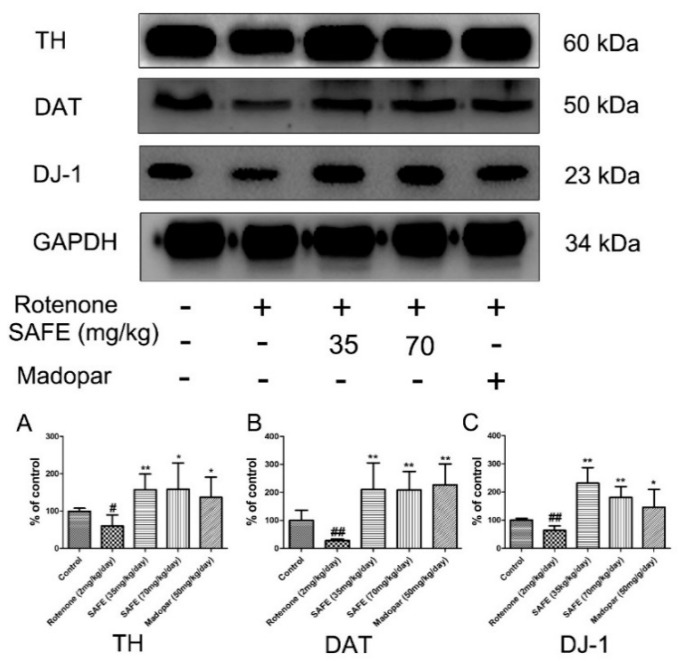
Western blot analysis. After 10 days of rotenone treatment, the levels of TH, DAT and DJ-1 were downregulated. After SAFE treatment, these decreases were reversed. (**A**) TH protein expression levels; (**B**) DAT protein expression levels; (**C**) DJ-1 protein expression levels. The parameters are presented as the mean ± SD, (**^#^** compared to control, **^#^** *p* < 0.05, **^##^** *p* < 0.01; * compared to rotenone, * *p* < 0.05, ** *p* < 0.01) (*n* = 4).

In the SN, western blot analysis showed that tyrosine hydroxylase (TH), dopamine transporter (DAT) and DJ-1 protein expression was significantly decreased in the rotenone-treated group compared to the control group. After the daily oral administration of either SAFE or Madopar^®^ for 24 days, all of these proteins showed significantly increased expression in comparison to the rotenone group (Figure 4). As shown in Figure 4A, low-dose SAFE treatment resulted in more significantly increased TH protein expression levels compared to the high-dose SAFE and Madopar^®^ groups (*p* < 0.01), high-dose SAFE-treated group and Madopar^®^-treated group (*p* < 0.05). All three of the treatment groups (low-dose SAFE, high-dose SAFE and Madopar^®^) showed significantly increased DAT expression compared to the rotenone group (Figure 4B, all *p* < 0.01). Regarding DJ-1 expression, both SAFE treatment groups showed significantly increased DJ-1 protein expression (*p* < 0.01 compared to the rotenone group) than the Madopar^®^-treated group (*p* < 0.05 compared to the rotenone group) (Figure 4C).

### 2.4. HPLC Analysis of DA and Its Metabolites

**Figure 5:**
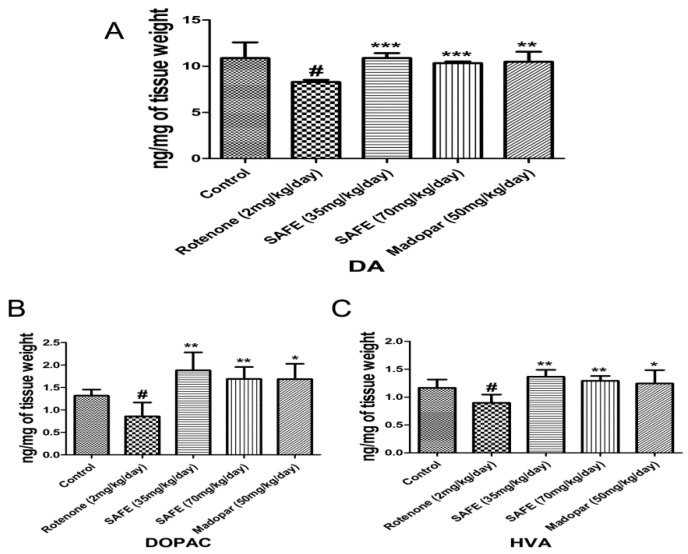
HPLC analysis of DA and its metabolites. Effects of SAFE on DA and its metabolites in the the striatum of rotenone-induced rat model of PD. (**A**) DA levels; (**B**) DOPAC levels; (**C**) HVA levels. The parameters are presented as the mean ± SD, (**^#^** compared to control, **^#^** *p* < 0.05; * compared to rotenone, * *p* < 0.05, ** *p* < 0.01, *** *p* < 0.001) (*n* = 4).

We measured the levels of DA and its metabolites dihydroxyphenyl acetic acid (DOPAC) and homovanillic acid (HVA) in the striatum using HPLC-based measurements. Rotenone injection caused significant decreases of DA and its metabolites DOPAC and HVA in the striatum compared to the control group (Figure 5A–C; *p* < 0.05). However, daily oral administration of low-dose SAFE, high-dose SAFE or Madopar^®^ increased the levels of DA (*p* < 0.001, *p* < 0.001 and *p* < 0.01, respectively, Figure 5A) as well as the levels of DOPAC (*p* < 0.01, *p* < 0.01 and *p* < 0.05, respectively, Figure 5B) and HVA (*p* < 0.01, *p* < 0.01 and *p* < 0.05, respectively, Figure 5C) compared to the rotenone group.

### 2.5. Mass Spectrometry Imaging (MSI) Analysis

**Figure 6:**
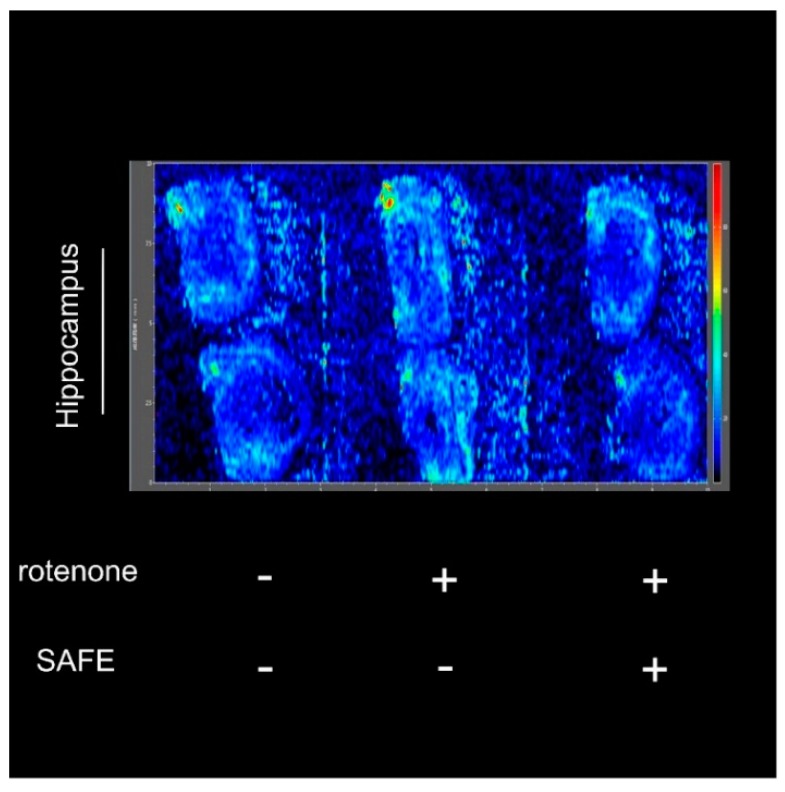
Imaging mass spectrometry analysis. After 10 days of rotenone treatment, increased Ach was detected in the hippocampus. After 24 days of low-dose SAFE (35 g/kg/day) treatment, the Ach levels were clearly decreased. Data are representative of three independent experiments with similar results (*n* = 3).

After measuring the changes in body weight and behavioral parameters, we confirmed the success of the rotenone-induced PD model and the efficiency of the drug treatment using the negative-ion scan mode (146.1146 *m*/*z*) to measure Ach levels. As shown in Figure 6, the hippocampal acetylcholine (Ach) levels in rotenone-treated rats were increased compared to control rats. However, after 24 days of low-dose SAFE treatment, the Ach content was decreased compared to the rotenone group (Figure 6).

### 2.6. Effects of SAFE on ECS Diffusion Parameters in the Striatum of Rats with Rotenone-Induced PD

**Figure 7:**
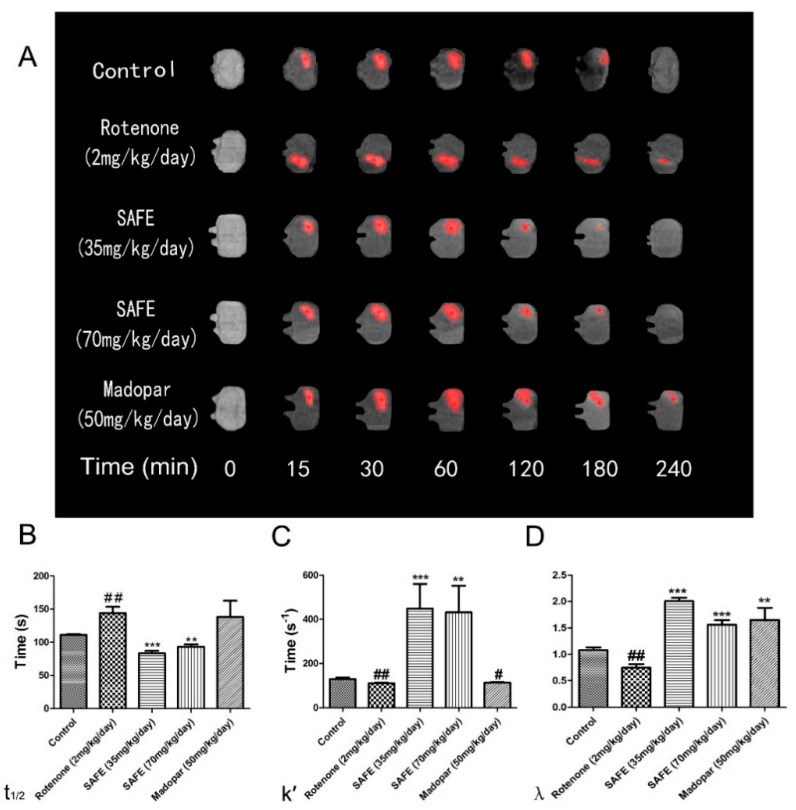
MRI tracer-based method measurement. Coronal views (**A**) of the MRI appearances of Gd-DTPA diffusion parameters (**B**) t_1/2_; (**C**) k′; (**D**) λ in the brain of rotenone-induced rat model of PD after SAFE treatment. The parameters are presented as the mean ± SD, (**^#^** compared to control, **^#^** *p* < 0.05, ^##^ *p* < 0.01; * compared to rotenone, ** *p* < 0.01, *** *p* < 0.001) (*n* = 4).

The free water diffusion coefficient (D) was calculated as 5.18 × 10^−4^ mm^2^/s in 0.3% agar gel at 37 °C [27]. In this study, we measured the effective diffusion coefficient (D*). The unilateral damage induced by rotenone in the striatum caused a significant extension in the *t*_1/2_ of Gd-DTPA, reduced the *k*′ and decreased the tortuosity (*λ* = $\sqrt{D/D*}$; all *p* < 0.01, Figure 7B–D). Both SAFE treatments resulted in significant shortening of *t*_1/2_ (*p* < 0.001 and *p* < 0.01, respectively, compared to the rotenone group, Figure 7B) and an increase in *k*′ (*p* < 0.001 and *p* < 0.01, respectively, Figure 7C) and *λ* (both *p* < 0.001, respectively, Figure 7D). Although Madopar^®^ treatment significantly increased the *λ* (*p* < 0.01, Figure 7D), it did not significantly increased the *k′* and shorten the *t*_1/2_ parameter (*p* > 0.05, Figure 7B,C). Figure 7A shows the elimination process of gadolinium-diethylene triamine pentaacetic acid (Gd-DTPA) in the striatum.

## 3. Discussion

The rotenone-induced PD model is associated with neuronal damage in the SN and striatum, which can manifest as marked deterioration in motor function, behavioral changes, loss of body weight and altered muscle morphology [28,29,30]. After 10 days of treatment with rotenone (2 mg/kg/day), treated animals developed symptoms of PD such as body weight loss, motor function impairment and behavioral changes.

Recent therapeutic advances in PD have revealed the promising role of flavonoids, which have been shown to ameliorate the loss of cognitive function by protecting susceptible neurons, maintain motor control to reduce motor complications, and sustain nigrostriatal integrity and functionality [31]. Findings by Gao X. [32] suggest that flavonoid intake may reduce PD risk, whereas Gao L. [33] reported that flavonoids from *Scutellaria* *baicalensis* Georgi possess neuroprotective properties. Gao L.’s report explored the effects of baicalein on motor behavioral deficits and showed that this compound significantly improved the abnormal behaviors in an MPTP-induced mouse model of PD.

In our study, SAFE treatment in rats with induced PD led to a marked recovery of body weight, motor function and behavioral changes. After 24 days of SAFE treatment, there were significant differences between the rotenone group and SAFE group with regard to body weight, rearing behavior and grip strength. The grip strength test was used to measure any alterations in the motor coordination. Both of tests were employed to assess behavioral deficits in the rats receiving subcutaneous or intravenous rotenone.

Rotenone treatment leads to nigrostriatal dopaminergic neurodegeneration, resulting in a loss of TH-positive neurons in the SN [34]. TH is a rate-limiting enzyme in the synthesis of DA as well as a transmitter and marker of dopaminergic neurons [34], and reduced levels of TH result in decreased levels of DA in the SN in patients with PD [35,36]. Released DA can be recycled back to the presynaptic terminal through the specific transporter DAT [37]. Dopaminergic neurons appear to be associated with the utilization of DAT; however, DAT does not mediate rotenone toxicity. DJ-1 protects DA neurons against mitochondrial dysfunction and oxidative stress through an autophagic pathway [38]. Pesticides and mitochondrial toxins are associated with mitochondrial dysfunction and oxidative stress, and in vivo rotenone exposure generates significant oxidative modification of DJ-1. These modifications are restricted to dopaminergic regions such as the SN, striatum, olfactory bulb and, to some extent, the cortex [39]. A previous study from our group identified DJ-1-binding compounds in safflower extract. By using a quartz crystal microbalance (QCM), we identified 5 DJ-1-binding compounds that have been utilized in Traditional Chinese Medicine (TCM) and possess anti-oxidant activities. SAFE included all 5 of these compounds, with K3R and AYB representing the most prevalent constituents. Thus, these two compounds were classified as the quality standard of SAFE.

In our experiment, HPLC analysis showed that the 30% ethanol elution of safflower contained K3R and AYB. Isolation of these compounds from safflower was identical to our earlier work, suggesting that this process has good reproducibility.

Western blot analysis is the one of the most commonly used methods to detect protein. In our study, we observed that the expression of TH, DAT and DJ-1 protein was significantly decreased in the rotenone-treated group; however, after SAFE treatment, these levels were restored. DAT exists in two forms: the glycosolated form (80 kDa) and non-glycosylated form (50 kDa). In this study, we observed that only non-glycosylated DAT was present in the brain tissue of rotenone-treated rats.

By HPLC, the present findings reveal a neuroprotective effect of SAFE in the rat rotenone model of PD. Rats treated with SAFE showed significantly increased DA levels as well as increased levels of the metabolites DOPAC and HVA in the striatum decreased by rotenone treatment.

As described above, DA and Ach are two primary factors directly involved in PD. Based on the western blot analysis and the HPLC results, we initially speculated that SAFE could positively influence the DA system. In normal situations, there is a balance between DA and Ach release in the brain. The significant loss of DA in the striatum plays a critical role in the pathogenesis of PD, which can result in low DA and high Ach levels. Thus, one approach to treat or cure PD is to increase the levels of DA while simultaneously decreasing the levels of Ach. To confirm the success of our rat model of PD as well as determine the efficacy of SAFE, we used MSI to measure the Ach levels.

MSI can indicate the anatomical distribution of a compound and is used for various applications in pharmaceutical research [40]. MSI is a powerful tool for directly determining the distribution of proteins, peptides, lipids, neurotransmitters, metabolites and drugs in neural tissue sections in situ and is thus an important analytical technique in neuroscience research. The imaging of biomolecules has provided new insights into multiple neurological diseases, including PD and AD [41]. Improper neuronal function related to abnormal concentrations of neurotransmitters in the brain has been associated with anxiety and depression, as well as diseases such as PD. Desorption electrospray ionization (DESI) mass spectrometry has also been used to image neurotransmitters [42]. In our experiment, the MSI results showed that rotenone caused an increase of Ach levels, while low-dose SAFE treatment (35 mg/kg/day) caused a clear decrease in hippocampal Ach levels.

The ECS is the microenvironment of the neurons and glia and is used as an important communication channel; this space includes ions, transmitters, metabolites, peptides, neurohormones and molecules of the extracellular matrix, all of which either directly or indirectly affect neuronal and glial cell functions [43,44]. The ECS is also essential for intercellular communication, nutrient and metabolite trafficking, and drug delivery [44]. Neurons and glia release a number of neuroactive substances that diffuse via the ECS to their targets located on other neurons and glial cells, which are frequently a far distance from the release sites, allowing for long distance extrasynaptic communication between cells [43]. Diffusion is an important transport mechanism for many substances introduced into the ECS of the brain [45]. The movement of neuroactive substances through the ECS of the CNS is the basic mechanism for extrasynaptic, or volume, transmission. In acute physiological as well as pathological states, diffusion in the ECS is affected by cell (especially glial) swelling, resulting from ionic and water shifts between intra- and extracellular compartments. In more slowly developing or chronic states, diffusion is affected primarily by structural changes of the tissue related to myelination and the rebuilding of fine astrocytic processes during development, lactation, aging, injury and degenerative diseases [46].The process of diffusion is sensitive to the ECS structure, and this sensitivity can be quantified by measuring the parameter that characterizes the diffusion process [47]. Macroscopic properties of this geometrically complex environment can be summarized by two parameters: the ECS volume fraction α and its tortuosity λ. The volume fraction determines what percentage of the total tissue volume is accessible to the diffusing molecules and is often referred to as porosity in the literature. Tortuosity describes the average hindrance of a complex medium relative to an obstacle-free medium [45].

PD is primarily characterized by the loss of dopaminergic neurons in the SNpc DA, also known as striatal depletion [48]. According to PD specialists, depletion of striatal DA may cause changes to the normal structure of the ECS. Awareness of molecular diffusion throughout the brain’s ECS has increased because of its potential role as a mediator of volume transmission and extrasynaptic communication.

The desire to measure diffusion properties of the brain ECS has led to the development of several techniques, including the MRI tracer-based method. MRI tracing is capable of determining water diffusion in the brain ECS [27]. Early studies using this tracer-based MRI method could quantitatively measure the local diffusion parameters of the brain ECS. Thus, the present study used this method to measure *t*_1/2_, *k*′ and *λ.* After rotenone treatment, rotenone-induced neuronal death, especially in the DA system, caused an extension of the *t*_1/2_ and a decrease of *k*′ and *λ_._* After SAFE treatment, the *t*_1/2_ was shortened, the *k*′ was increased, and *λ* recovered to baseline levels. Our study also showed that compared to SAFE, Madopar^®^ was unable to improve motor behavior, body weight loss, *t*_1/2_ and *k*′. These results suggest that SAFE is superior to Madopar^®^ in improving clinical symptoms and protecting dopaminergic neurons in rotenone-induced PD rats.

Throughout the experiment, we observed that a lower dose of SAFE (35 mg/kg/day) resulted in more neuroprotective effects than a higher dose of SAFE (70 mg/kg/day) with regard to changes in body weight, rearing behavior and grip strength. The low dose of SAFE was also more effective in increasing the expression of TH compared to the high dose of SAFE. There also appeared to be a correlation between the Ach levels and the neuroprotective effects of SAFE at either dose. However, additional studies focusing on the effects of SAFE on Ach are required.

## 4. Materials and Methods

### 4.1. Reagents and Animals

Rotenone was purchased from Sigma Aldrich (St. Louis. MO, USA). Safflower was purchased from Beijing San-He Pharmaceutical Co., Ltd. (Beijing, China). Madopar^®^ was purchased from Roche Pharmaceutical Ltd. (Pudong, Shanghai, China). Healthy male Sprague-Dawley (SD) rats weighing 280–320 g were purchased from Beijing HuaFuKang Bioscience Co. Ltd. (Beijing, China) with the confirmation number SCXK (Jing) 2014-0004. The animal study was approved by the Institutional Animal Care and Use Committee at Peking University (permit No. LA2016245), and all animal procedures were performed according to the IACUC policy. All efforts were made to minimize animal suffering and reduce the number of animals used. All rats were housed under standardized housing conditions (12/12 h light/dark cycle; temperature, 22 ± 2 °C; relative humidity, 50% ± 5%) and were provided food and water adlibitum.

### 4.2. Herbal Extract and Qualitative Analyses of the SAFE Components

**Figure 8:**
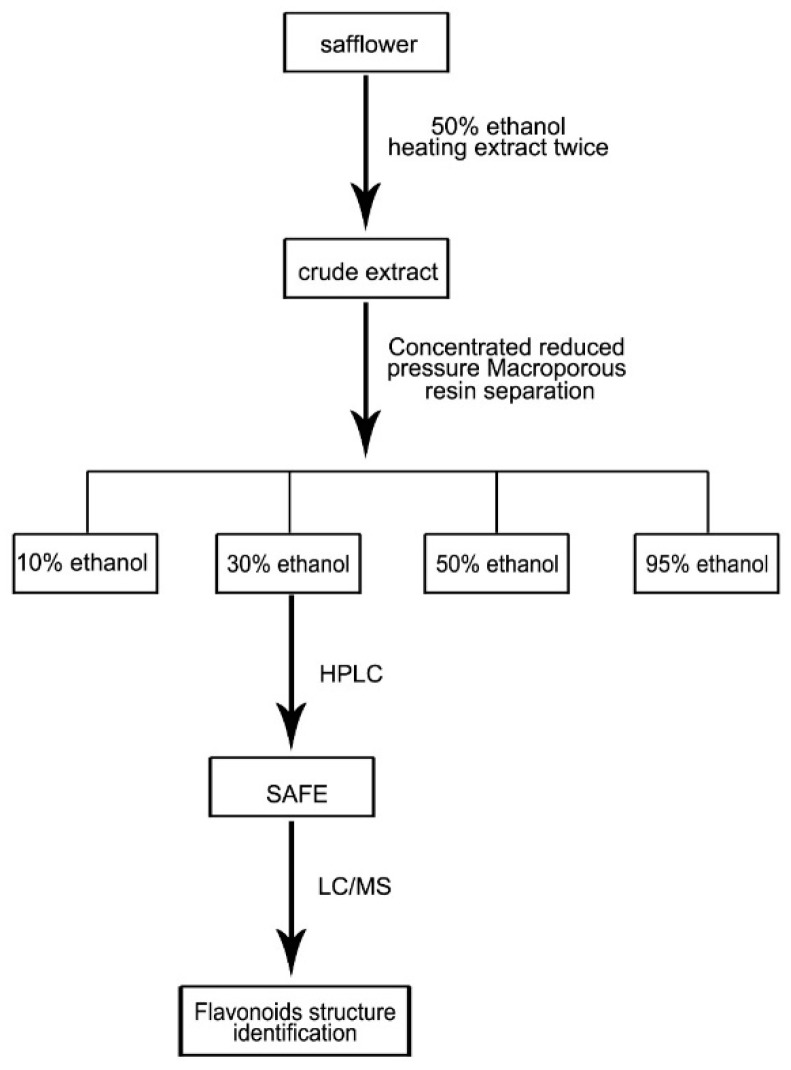
Extraction of SAFE route map.

Based on a previous report [26] and minor modifications, 8 kg of safflower were soaked in 50% ethanol (8 L/kg) for 2–3 h, followed by heat extraction under reflux twice, 1 h for each cycle. The extracts were combined, concentrated in vacuum, and diluted with de-ionized water to produce a thin extract at a concentration of 1 g/mL. The macroporous resin separation method was used to elute the extract with 10%, 30%, 50% and 95% ethanol (Figure 8), and the content was determined using HPLC and LC/MS analyses. LC/MS conditions: HPLC‑DAD‑MS^n^ analysis was performed on a Shimadzu LC-20A instrument (Kyoto, Japan) coupled with an ion-trap-time-of-flight mass spectrometer (Shimadzu Corp., Kyoto, Japan) via an electrospray ionization interface. Chromatographic separation was performed on an Alltima-C18 column (5 μm, 250 mm× 4.6 mm, Grace Alltech Corp., Columbia, MD, USA) protected with an Eclipse Plus C_18_ guard column (5 μm, 4.6 mm × 12.5 mm, Agilent Incorp., Santa Clara, CA, USA). The column was maintained at 30 °C and the flow rate was 1.0000 mL/min. The mobile phase consisted of water containing 0.3% formic acid (A) and acetonitrile:methanol = 9:1 (B) following the gradient program: 0 min: 90% (A), 10% (B); 5 min: 86% (A), 14% (B); 25 min: 83% (A), 17% (B); 35 min: 82% (A), 18% (B); 65 min: 70% (A), 30% (B); 75 min: 70% (A), 30% (B). The post-column splitting ratio to source was 1:4. The ESI source was operated in the negative ion and positive ion detection mode. Other parameters were as follows: curved desolvation line and heat block temperature, 200 °C; nebulizing nitrogen gas flow: 1.5 L/min; interface voltage: (+), 4.5 kV; (−), −3.5 kV; detector voltage: 1.70 kV; mass range: MS, *m/z* 130–1500; MS^2^ and MS^3^, *m/z* 50–1500; Data were analyzed using LCMS solution Version 3.60 software (Shimadzu, Kyoto, Japan).

### 4.3. Animal Treatment

Prior to the experimental procedure, the rats were acclimatized for one week. After one week, the animals were randomly divided into two groups: the control (*n* = 10) and rotenone-treated groups (*n* = 92). The rotenone solution was prepared (2 mg/mL of rotenone in 98% sunflower oil, 2% DMSO), and rats in the rotenone-treated group were subcutaneously injected with 2 mg/kg/day of rotenone solution once per day for 10 days. Rats in the control group were injected with the same volume of vehicle.

### 4.4. Screening for Motor Impairments

Twenty four h after the final rotenone injection (day 10), rats were screened for motor impairments using the rearing behavior test and grip strength test. Testing the rearing behavior was performed as previously described [5]. When placed in a clear cylinder, rats will engage in exploratory behavior, including rearing. During rearing behavior, the forelimbs will contact the wall of the cylinder. For this test, the rat was placed in a clear plexiglass cylinder (height = 30 cm, diameter = 20 cm) for 5 min. To assess grip strength, rats were lifted gently by the tail and allowed to grasp a rigid bar attached to a force transducer and digital display unit. When the first signs of active grasp were observed, the rats are pulled upward slowly by the tail with increasing firmness until their grasp was overcome. The peak force was recorded as the maximum grip strength. The test was repeated 3–5 times/limb, and the maximum grip strength per trial was included in the statistical analysis. After 10 days, the rats in the rotenone-treated group were randomly divided into four groups according to their behavioral test and body weight: the Madopar^®^ group (50 mg/kg/day), the SAFE low-dosage group (35 mg/kg/day), the SAFE high-dosage group (70 mg/kg/day) and model group. The secondary treatment groups were administered their respective doses by oral gavage once per day. After 24 days, rats were anesthetized with 10% chloral hydrate solution (0.35 mL/100 g, i.p.), sacrificed and perfused, after which the rats’ brains were removed no longer than 30 s post-mortem and frozen on powdered dry ice before transferring to a −80 °C freezer. The SN tissue was used for western blotting, and the striatum was used for HPLC.

### 4.5. Western Blot Analysis

For western blot analysis, the SN tissues were homogenized on ice in RIPA buffer and centrifuged at 10,000 rpm for 30 min to isolate the supernatant. The protein concentration was determined using a BCA protein assay (Pierce, Jinshan, Shanghai, China). Samples were run on 12.5% SDS-PAGE gels with a total volume of 30 μg of protein loaded per lane. The separated proteins were transferred to a PVDF membrane using a semidry transfer system (Bio-Rad, Hercules, CA, USA). After blocking with 5% nonfat milk in TBST, the membranes were incubated overnight at 4 °C with the following primary antibodies: TH anti-rabbit polyclonal antibody (1:1000), DAT anti-rabbit polyclonal antibody (1:500), DJ-1 anti-mouse monoclonal antibody (1:1000) and GAPDH anti-rabbit polyclonal antibody (1:1000) (Santa Cruz Biotechnology, Santa Cruz, CA, USA). After primary antibody incubation, membranes were washed with TBST and incubated with either anti-rabbit (1:2000) or anti-mouse secondary antibody (1:5000), after which the membranes were washed with TBST. Blots were scanned as grayscale images and quantified using Quantity One software (version 4.6.2, Hercules, CA, USA). The protein levels were normalized against the levels of GAPDH, and the optical density of each band was quantified [8,49].

### 4.6. HPLC Analysis of DA and Its Metabolites

The levels of DA and its metabolites DOPAC and HVA were analyzed using an electrochemical (EC) detector (BAS LC-4B, BASi Corp., West Lafayette, IN, USA) as previously described [50,51]. The mobile phase was sodium citrate buffer (85 mM citric acid, 100 mM anhydrous sodium acetate and 0.2 mM Na_2_EDTA; pH 3.68). The flow rate was 1.2 mL/min at 25 °C in the reversed phase column. After centrifugation (4 °C, 15,000× *g*, 20 min), 20 μL of striatum tissue homogenate supernatant was injected directly into the HPLC system. Data were calibrated with an external standard. The levels of DA and its metabolites were calculated and expressed as ng/mg tissue weight.

### 4.7. Mass Spectrometry Imaging (MSI) Analysis

To collect respective tissue samples as well as obtain tissue slices for MSI analysis, male SD rats (typically 280–320 g) were deeply anesthetized and decapitated after scarification approximately 60 min after the last administration of SAFE (35 g/kg/day). The brain was removed a maximum of 30 s post-mortem and frozen on powdered dry ice before transferring to a −80 °C freezer [52,53]. Frozen hippocampus tissue was cut to 6-μm slices on a cryostat microtome and was used for MSI analysis with an AFAI-MSI imaging platform system [54,55,56,57]. Air flow-assisted desorption electrospray ionization mass spectrometry imaging (AFADESI-MSI) was used to measure the levels of the neurotransmitter Ach. To minimize oxidation, tissue sections were analyzed within the shortest possible time after sectioning, even if stored at −80 °C.

### 4.8. The Measurement of ECS Diffusion Parameters with the MRI Tracer-Based Method

Previous studies using the tracer-based MRI method have quantitatively measured the local diffusion parameters of the brain ECS [27], as previously described by Li et al. [58]. After the final drug administration, we microinjected 2 μL Gd-DTPA solution (10 mM) into the rat striatum over a period of five minutes, and waited five more minutes to avoid dorsal reflux along the needle track. The anesthetized rat was placed in the prone position and scanned with a T1-weighted three-dimensional magnetization prepared-rapid acquisition gradient echo (T1 3D MP-RAGE) sequence in a 3.0 Tesla MRI systems machine (Magnetom Trio, Siemens Medical Solutions, Erlangen, Germany). Based on the Gd-DTPA injection time, repeated scans were performed for each subject at different time intervals (15 min and 30 min and 1,2,3 and 4 h post-injection).

Calculation of the diffusion parameters was performed as previously described [25,27,58]. The imaging sequence and parameters were identical to those used in the MRI scan protocol. A MATLAB-based software program (NanoDetect, version 1.3, MathWorks, Beijing, China) was developed to co-register the MR images of the same rat before and after the injection [25,27,58]. Then, the program identified areas that were enhanced by Gd-DTPA injection according to the modified diffusion equation based on the linear relationship between the MRI signal increment and the average Gd-DTPA concentration in brain tissue; the T1 3D MP-RAGE sequence was used for the in vivo measurement of the Gd-DTPA concentration in real-time neuroimaging at 3.0 T [28,58,59]. The diffusion parameters *t*_1/2_, *k*′ and *λ* were calculated using the MATLAB-based software at each time point post-injection.

### 4.9. Statistics

The parameters are presented as the mean ± SD. The significance of the differences between groups was evaluated using one-way analysis of variance (ANOVA) with the Student-Newman-Keuls post hoc test. Differences were considered to be statistically significant when *p* < 0.05.

## 5. Conclusions

In conclusion, a rodent model of PD was generated successfully through the subcutaneous injection of rotenone, which led to neurodegeneration as previously reported. This model was employed to understand the pathogenesis of PD and develop novel neuroprotective therapies. In this study, K3R and AYB comprised the quality standard of the safflower extract and demonstrated the neuroprotective properties of flavonoids. Oral administration of two doses of SAFE (35 mg/kg/day and 70 mg/kg/day) was observed to provide a significant neuroprotective effect in the rotenone-induced rodent model of PD. SAFE also demonstrated the potential to inhibit apoptosis triggered by neurotoxic species and promote neuronal survival, as shown by the increase in body weight, recovery from motor function impairment and behavioral changes following treatment. Furthermore, SAFE affected the DA system, shortened the *t*_1/2_ and increased the *λ* and *k*′ SAFE also reduced hippocampal Ach levels, although the mechanism involved requires further study.

## Figures and Tables

**Table 1:** Representative compounds identified in the SAFE by HPLC-ESI-IT-TOF-MS^n^ analysis.

No.	*t*_R_ (min)	Meas. *m/z* ([M − H]^−^)	Diff. (ppm)	Formula	Characterisc Fragment Ions	Identification
1 *	9.122	611.1609	1.47	C_27_H_32_O_16_	MS^2^: 593.1455(0.83), C_27_H_29_O_15_; 521.1235(1.86), C_24_H_25_O_13_; 503.1153(1.27), C_24_H_23_O_12_; 491.1148(100), C_23_H_23_O_12_; 485.1192(0.19), C_24_H_21_O_11_; 473.1047(9.40), C_23_H_21_O_11_; 461.1045(0.76), C_22_H_21_O_11_; 421.1120(4.41), C_20_H_21_O_10_; 403.1088(28.95), C_20_H_19_O_9_; 385.0892(6.81), C_20_H_17_O_8_; 325.068332.65), C_18_H_13_O_6_; 313.0699(5.68), C_17_H_13_O_6_; 283.0594(11.67), C_16_H_11_O_5_. MS^3^(491.1148): 473.1105(82.37), C_23_H_21_O_11_; 353.0593(45.23), C_19_H_13_O_7_; 323.0514(61.48), C_18_H_11_O_6_; 301.0705(62.80), C_16_H_13_O_6_; 283.0625(100), C_16_H_11_O_5_.	hydroxysafflor yellow A (HSYA)
**4**	19.348	609.1432	−3.61	C_27_H_30_O_16_	MS^2^: 301.0329(100), C_15_H_9_O_7_; 300.0262(7.36), C_15_H_8_O_7_. MS^3^(300.0265): 271.0292(7.98), C_14_H_7_O_6_; 255.0255(100), C_14_H_7_O_5_; 244.0250(80.91), C_12_H_5_O_6_; 173.0889(10.06), C_11_H_9_O_2_.	6-hydroxykaempferol 3-*O*-β-d-rutinoside (6H3R) or quercetin 3-*O*-rutinoside (rutin)
16 *	33.482	593.1502	−2.87	C_27_H_30_O_15_	MS^2^: 285.0404(100), C_15_H_9_O_6_, [M – H − rutinosyl]^−^. MS^3^(285.0404): 257.0422(100), C_14_H_9_O_5_; 239.0377(36.25), C_14_H_7_O_4_; 267.0273(51.50), C_15_H_7_O_5_; 229.05410(74.95), C_13_H_9_O_4_; 163.0080(49.24), C_8_H_3_O_4_; 150.9977 (15.98), C_7_H_3_O_4_, ^1,3^A^−^.	kaempferol-3-*O*-rutinoside (K3R)
**17**	36.083	623.1614	−2.73	C_28_H_32_O_16_	MS^2^: 608.1311(0.66), C_27_H_28_O_16_, [M − H − CH_3_]^•−^; 477.0929(0.13), C_22_H_21_O_12_, [M – H − 146 (C_6_H_10_O_4_, methylpenstosyl)]^−^; 357.0626(1.70), C_18_H_14_O_8_; 315.0503(100), C_16_H_11_O_7_, [aglycon − H]^−^; 300.0257(37.43), C_15_H_8_O_7_; 271.0221(16.31), C_14_H_7_O_6_; 255.0306(5.67), C_14_H_7_O_5_. MS^3^(315.0503): 255.0327(3.63), C_14_H_7_O_5_; 181.0147(0.19), C_8_H_5_O_5_.	isorhamnetin methylpentosyl hexoside
**18** *	37.790	1043.2646	−2.68	C_48_H_52_O_26_	MS^2^: 1025.2493, C_48_H_49_O_25_; 923.2204, C_44_H_4__3_O_22_; 449.1065, C_2__1_H_21_O_11_. MS^3^(449.1065): 431.0942, C_2__1_H_1__9_O_10_; 329.0574, C_13_H_1__3_O_10_; 311.0489,C_13_H_11_O_9_; 299.0527, C_16_H_11_O_6_; 287.0534, C_15_H_1__1_O_6_; 261.0660, C_10_H_1__3_O_8_; 259.0604, C_14_H_1__1_O_5_; 241.0495, C_14_H_9_O_4_; 207.0557, C_7_H_11_O_7_; 178.9990, C_8_H_3_O_5_; 153.0181, C_7_H_5_O_4_.	anhydrosafflor yellow B (AYB)
**23**	42.513	449.1077	−2.67	C_21_H_22_O_11_	MS^2^: 287.0553, C_15_H_11_O_6_. MS^3^(287.0553): 181.0257, C_8_H_5_O_5_; 153.0119, C_7_H_5_O_4_; 119.0573, C_8_H_7_O.	neocarthamin or its isomer

* Confirmed by comparison with reference compounds.
